# Experimental inoculation of *Treponema pedis* T A4 failed to induce ear necrosis in pigs

**DOI:** 10.1186/s40813-017-0073-2

**Published:** 2017-12-19

**Authors:** Frida Karlsson, Anna Rosander, Claes Fellström, Annette Backhans

**Affiliations:** 1 Farm and Animal Health, Klustervägen 11, SE-590 76 Vreta Kloster, Sweden; 2 Department of Biomedical Sciences and Veterinary Public Health, Swedish University of Agricultural Sciences, SE-75007 Uppsala, Sweden; 3 Department of Clinical Sciences, Swedish University of Agricultural Sciences, SE-75007 Uppsala, Sweden

**Keywords:** Pig, Ear necrosis, *Treponema*, IgG, Experimental infection

## Abstract

Ear necrosis is a syndrome affecting pigs shortly after weaning and is regarded as an animal welfare issue. The etiology is unknown but *Treponema* spp., predominantly *Treponema pedis*, are commonly detected in the lesions. Oral treponemes have been suggested as source of infection, transferred by biting and licking behavior. In this study, five pigs were intradermally inoculated with *Treponema pedis* strain T A4 with the aim of investigating if this strain would induce ear lesions. Three pigs served as controls. The inoculation was repeated after 29 days, and the study continued for 56 days. Serum samples were collected throughout the study and analyzed by ELISA for IgG antibodies towards *T. pedis* T A4 lysate. Skin biopsies were taken from the inoculation area at the end of the study. Gingival samples were collected and cultivated for treponemes, for comparison to the inoculation strain and to follow colonisation. The challenged pigs did not develop any clinical signs of infection and no spirochetes were detected in sections from skin biopsies. The number of *Treponema*-positive gingival samples increased during the study. In the challenge group, IgG towards the bacterial lysate peaked 7 days after each inoculation and decreased rapidly hereafter. In the control group a weak IgG response was observed after the second inoculation, possibly caused by the oral treponemes.

## Background

Ear necrosis is observed in pigs after weaning or during the early grower period, and may occur as an outbreak. The lesions involve the ventral margins or the tip of the ear and are usually bilateral [1, 2]. Severe cases are characterized by hyperemia, edema, exudation, ulceration and necrosis that may spread to involve a large part of the ear [3], and eventually the ear may be sloughed off [1]. We previously showed that *Treponema* spp., predominantly *T. pedis,* were common and abundant in both shoulder ulcers and ear necrosis [4, 5], and similar results were presented by Clegg et al. [6]. The detection of oral treponemes closely related to ulcer treponemes led us to hypothesize that treponemes are transferred to the skin by biting and licking behavior [4]. However, further studies are needed to reveal if treponemes are involved with the aetiology as the primary cause of the syndrome or if they are secondary invaders of the skin ulcers. The aim of this study was to test if inoculation with *T. pedis* T A4 could induce lesions of ear necrosis in pigs, as evaluated by clinical and histopathological examination. The immune response was analysed and gingival samples were continuously checked for treponemes to study the colonization of the gingiva and to enable comparison with the inoculation strain.

## Methods

The study was performed at the department of Clinical Sciences at the Swedish University of Agricultural Sciences, Uppsala, in research animal facilities (see Declarations). The study included eight pigs (A-H) of Yorkshire/Hampshire crossbreed from two litters born by Yorkshire gilts. Two males and two females from each litter were randomly selected at four weeks, when weaned, and were allocated to the treatment group (*n* = 5, A-E) or the control group (*n* = 3, F-H). During the acclimatization period of one week a wet bandage was applied to one ear of each animal to keep the skin moist. The animals were individually housed in 3m^2^ pens with concrete floor, infrared lamps and bedding of wooden shavings and straw. Water was given ad libitum. A commercial diet (Solo 330 P SK 25 kg, Lantmännen) and hay was fed twice daily. At day 0, the pigs were 5 weeks and their weight varied between 8 and 11 kg. The bacterial strain, *T. pedis*, T A4, previously isolated from a pig with ear necrosis [7], was thawed and recultured as previously described [8] for two passages before inoculation. After 3–5 days the cultures were fully grown and in the log phase, based on visual estimation of density and phase-contrast microscopy of the spirochetes shape and motility. Broth cultures were centrifuged and the bacterial pellets were washed twice with isotonic saline, centrifuged and diluted with isotonic saline to a density of ≥5 Mc Farland Standards. The animals (A-H) were anaesthetized according to a protocol by Malavasi et al. [9]*.* After disinfection with ethanol, both ears were injected with 0.5 ml solution, with an estimate of 10^9^ bacteria (challenge pigs, A-E) or isotonic saline (control pigs, F-H), evenly distributed at four sites, approximately ¼ in on the earlobe and 0.5 cm from the ventral margin, on both sides of the earlobes. On pigs C, E, and F a blunt trauma was made by shutting a forceps on five sites along the margin of the ear. The wet bandage was kept for one week after injection. Pig B was excluded from the study from day 7 due to fainting and vomiting. At day 29 the inoculation was repeated. Thereafter the pigs were grouped two and two in the pens (A + C, D + E, F + G), except for pig H that was individually housed. The ears and the skin of the pigs were examined daily. Serum samples were collected from *vena jugularis externa* at days 0, 7, 11, 14, 21, 29, 35, 42, 49, 56. Gingival samples were taken with cottons swabs. At day 56 biopsies were collected from each ear from all pigs under anesthesia, and the pigs were euthanized by intravenous injection with 140 mg/kg pentobarbital sodium and phenytoin sodium (Euthasol® vet. Virbac Animal Health). After fixation in formalin the biopsies were embedded in paraffin and sections of 5–7 μm were cut and stained with hematoxyline and eosin (HE) and Warthin-Starry silver staining (W-S). Gingival samples were investigated by phase contrast microscopy and inoculated and cultured as previously described [8]. Pure spirochetal isolates were analysed by PCR and subsequent sequencing of the 16S ribosomal RNA-tRNA-Ile intergenic spacer region (ISR2) and 16S ribosomal RNA genes [7, 10]. Sequences were processed in CLC Main Workbench 6.7 (CLC Bio) and the megablast algorithm BLAST was used for homology searches [11].

For the enzyme-linked immunosorbant assay (ELISA)*, T. pedis* T A4 lysate was prepared by washing bacterial pellets from eight 10 ml cultures three times with isotonic saline, suspended in 8 ml of isotonic saline and subjected to ultrasonic treatment using a horn-type sonicator (Vibra-cell VC-505; Sonics & Materials Inc., Newton, NJ, USA) at a frequency of 20 kHz. The cell lysate was sterile filtered using 0.2 μm syringe filters (Sartorius, Goettingen, Germany) and quantified with Picodrop Microliter UV/Vis Spectrophotometer. Assays were performed as described by Rosander et al., 2011 [12], with the following modifications: microplates (C96 Polysorp, Nunc-immuno plate) were coated at 4 °C over night (> 16 h) with the lysate at concentrations of 5 μg/ml in 100 μl 50 mM sodium carbonate and washed three times before blocking with PBS-T (phosphate buffered saline pH 7.4 with 0.05% Tween 20) for 30–45 min at room temperature. Serum samples, diluted 1:100 in PBS-T, were added in duplicate and pig IgG was detected with anti-pig IgG (whole molecule) peroxidase conjugate antibodies developed in rabbit (Sigma), diluted 1:20,000, after which wells were washed four times with PBS-T. Optical density was measured at 450 nm after the addition of 1 mM tetramethylbenzidine and 0.006% H_2_O_2_ in 0.1 M potassium citrate pH 4.25, incubating the plates for 10 min at room temperature, and stopping the reaction by addition of 50 μl 10% sulfuric acid per well. The significance of difference between groups was assessed by two-sample t-test using Minitab 17 Statistical software (Minitab Inc., Harrisburg, PA, USA).

## Results

**Fig. 1:**
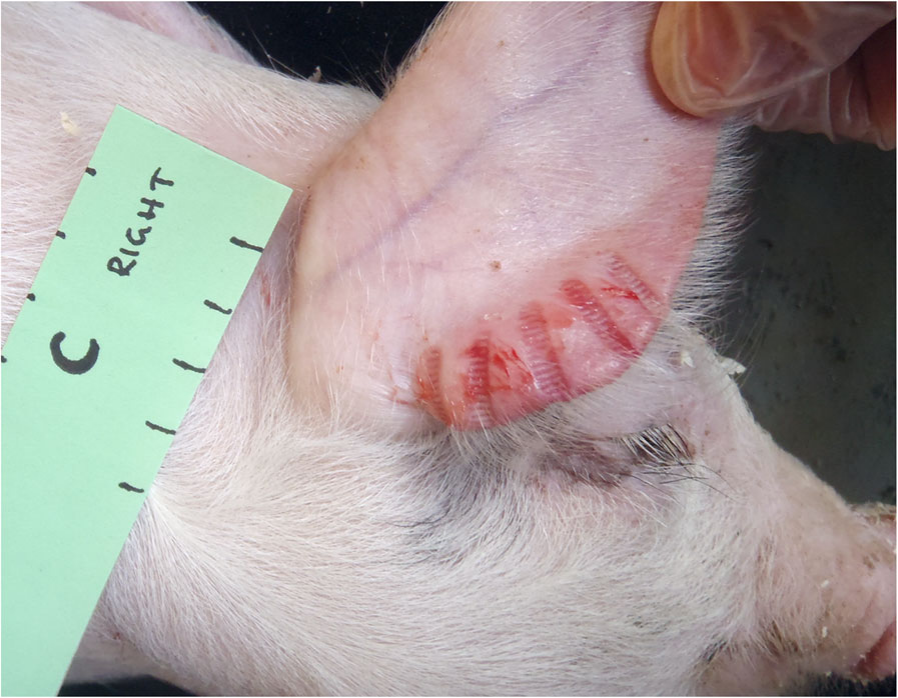
Pig C, day 0

No clinical signs of infection could be detected. After inoculation, a mild erythema was observed in the area of inoculation in both challenge and control pigs (Fig. 1). It decreased gradually and had disappeared within ten days. On the ears that had been exposed to a blunt trauma a brown scab could be noted up to 14 days. No differences were noted between challenged and control pigs. HE stained sections from challenged and control pigs at day 56 showed focal, mild perivascular inflammatory changes in the dermis with eosinophils and lymphocytes. No spirochetes were detected in any of the W-S sections. The results from the microscopic observations of gingival samples are shown in Table 1.

**Table 1:** Detection of *Treponema*-like spirochetes from gingival samples of challenge pigs (A-E) and control pigs (F-H) by phase contrast microscopy of FAB + A^a^ broth cultures

Day	0	7	14	21	29	35	42	49	56
Age	5 w^b^	6 w	7 w	8 w	9 w	10 w	11 w	12 w	13 w
Pig A	–	–	–	–	+	–	–	+	–
Pig B	–	nt^c^	nt	nt	nt	nt	nt	nt	–
Pig C	–	–	+	–	+	+	+	+	+
Pig D	+	–	–	–	+	–	+	+	+
Pig E	–	–	–	–	–	+	+	+	–
Pig F	–	–	+	–	+	+	+	+	+
Pig G	–	+	–	–	+	–	+	+	–
Pig H	–	–	–	–	+	–	–	–	+

^a^FAB + A = Fastidious anaerobe broth with addition of glucose, thiamine pyrophosphate, volatile fatty acids, fetal calf serum, enrofloxacin and rifampicin [8]

^b^w = weeks^c^ nt = not tested

Four isolates were obtained, from challenge pigs C and E, and from control pigs F and H.

**Fig. 2:**
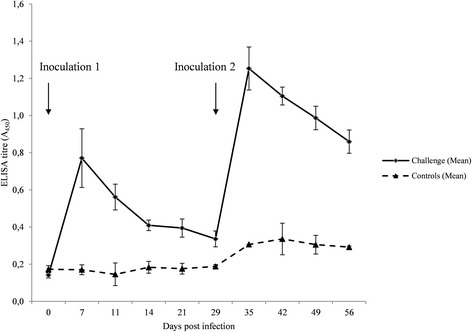
Mean ELISA titres of serum IgG to *T. pedis* T A4 lysate for the pigs in challenge and control group throughout the study. Standard deviations are shown as whiskers. Inoculation time points as arrows

The 316 nucleotides long ISR2 sequences of these four isolates were identical, and the homology search showed that they matched most closely (98% homology) to two strains of *T. pedis* previously isolated from pig ear necrosis; isoE1186 (accession no KC619314) and the challenge strain T A4 (CP004120), and one from pig gingiva; T M1 (KC619311). The 16S rRNA sequences of the same isolates matched most closely (99% homology) to strains T A4 (CP004120) and T M1 (FJ805835), same as above, and strain G179 (AF363634), isolated from a case of ovine foot rot. On day 7 the challenge pigs had developed an IgG response towards the *T. pedis* T A4 lysate, that thereafter decreased steadily until re-inoculation on day 29, when a similar rapid increase in antibody response was followed by a decrease (Fig. 2). In the control group, a weak response developed from day 35, and mean absorbance values were significantly higher at the end of the study on day 56 (M = 0.29, SD = 0.00958) than on day 0 (M = 0.17, SD = 0.019) (two-sample t-test, *p* = 0.011).

## Discussion

To our knowledge, this is the first attempt to induce ear necrosis in pigs using a pure *Treponema* spp. culture. We used *T. pedis* as our previous studies indicated this species as the main phylotype in both ear necrosis and shoulder ulcers [4, 5]. Also, *T. pedis* is part of the treponemal consortium associated with bovine digital dermatitis (BDD) [13, 14]. The strain T A4 was selected as it originates from an outbreak of severe ear necrosis [7] and as several putative virulence related genes have been identified in its genome [15]. However, in this study T A4 did not cause any skin lesions. The reason for this negative outcome could be that *T.pedis* is not the aetiological agent of ear necrosis. However there might be other reasons. For example, the amount of bacteria could have been insufficient, similarly to a murine model for BDD where lesion development was demonstrated to be dose dependent [16]. We applied a wet bandage to one ear of each animal to mimic the only successful reproduction of BDD-like lesions in dairy cattle using one pure treponemal strain [17]. However, to keep the bandages on the ears proved very difficult and they had to be removed after one week. For future studies we suggest a development of this approach and a longer application period. In our previous studies *T. pedis* was the main phylotype in porcine skin ulcers, but a great diversity of treponemal phylotypes was revealed [4, 5]. In older studies, ulcers were successfully reproduced in healthy animals using scraping material from ulcers containing motile spirochaetes [18, 19]. Maybe a consortium of different treponemal species is required to cause ulcers also in the pig. One approach for future challenge studies is to include a mixture of different treponemal species, possibly in combination with other putative pathogens like staphylococci or streptococci [1, 20]. The etiology of ear necrosis is suggested to involve other factors like ear biting and stress mediated by weaning, mixing of pigs and high stocking rates [20]. Thus, our experimental design with individually housed animals, chosen to avoid contamination of the inoculated area by oral treponemes through biting or licking behavior, may have prevented ear necrosis instead of inducing the disease.

There was a significant IgG response already 7 days post infection in the challenge group, but this response was short and declined rapidly. A re-inoculation boosted the response but as the study ended 26 days after the second inoculation we don’t know if the levels would drop even further. In cattle the immune response to *T. phagedenis*-like spirochetes has been of short duration [21]. Our control group did show a weak IgG response at day 35, one week after re-inoculation of the challenge group. Interestingly, at that time most pigs in the study were colonized by gingival treponemes, why one could speculate that transmission of those could have occurred by biting or licking. However, also the single-housed pig showed this weak response. Presumably, colonization of gingiva is initiated already during the suckling period and, also, it cannot be ruled out that some low-dose transmission occurred between the pens and/or pigs during daily care. Our findings that treponemes 1) gradually colonized the pigs gingiva around the same age period as when ear necrosis usually occur, and 2) were identified as *T. pedis* closely related to strains isolated from pig ulcers, are in line with our previous suggestion that the mouth is the reservoir for the ulcer treponemes [4].

## Conclusions

In the presented model *T. pedis* T A4 failed to cause skin lesions similar to those of ear necrosis. IgG antibodies against T A4 lysate developed quickly after inoculation and then rapidly declined.
